# Real‐World Effectiveness and Safety of Damoctocog Alfa Pegol in Severe and Nonsevere Patients With Hemophilia A From the Prospective, Multinational, Ongoing HEM‐POWR Study

**DOI:** 10.1111/ejh.70026

**Published:** 2025-10-22

**Authors:** Mark T. Reding, María Teresa Alvarez Román, Giancarlo Castaman, Maissaa Janbain, Tadashi Matsushita, Karina Meijer, Kathrin Schmidt, Johannes Oldenburg

**Affiliations:** ^1^ Center for Bleeding and Clotting Disorders University of Minnesota Medical Center Minneapolis Minnesota USA; ^2^ Thrombosis and Haemostasis Unit Hospital Universitario La Paz Madrid Spain; ^3^ Department of Oncology, Center for Bleeding Disorders and Coagulation Careggi University Hospital Florence Italy; ^4^ Deming Department of Internal Medicine, Section of Hematology and Medical Oncology Tulane School of Medicine New Orleans Louisiana USA; ^5^ Department of Transfusion Medicine Nagoya University Hospital Nagoya Japan; ^6^ Department of Hematology University Medical Center Groningen Groningen the Netherlands; ^7^ OS Operations Bayer Berlin Germany; ^8^ Institute of Experimental Hematology and Transfusion Medicine University Hospital Bonn, Medical Faculty, University of Bonn Bonn Germany

**Keywords:** bleeding, extended half‐life, hemophilia A, inhibitors, nonsevere, real‐world, safety

## Abstract

**Objectives:**

To assess the effectiveness and safety of damoctocog alfa pegol in patients with severe and nonsevere hemophilia A in the fifth interim analysis of the ongoing HEM‐POWR study.

**Methods:**

HEM‐POWR (NCT03932201) is a multinational, Phase 4, prospective observational study. The key objectives were the annualized bleeding rate (ABR) and adverse events.

**Results:**

At data cutoff (July 8, 2024), the safety analysis set and full analysis set (FAS) included 370 and 270 patients, respectively. In the modified FAS (patients with ≥ 90 days of bleed data), the mean (standard deviation; SD) ABR for total bleeds was 2.8 (5.9) 12 months prior to damoctocog alfa pegol initiation with the previous FVIII product and 2.1 (4.8) during the observation period. Bleed protection with damoctocog alfa pegol was maintained across disease severity, age group, BMI group, dosing regimen, and patient inhibitor history. One patient died due to spinal cord ischemia unrelated to the study drug. One patient developed a transient low‐titer inhibitor, which resolved without clinical consequence.

**Conclusions:**

This updated analysis further demonstrated the effectiveness, acceptable safety profile, and tolerability of damoctocog alfa pegol for previously treated patients with severe and nonsevere hemophilia A from adolescence to older age in a real‐world setting.

## Graphical abstract

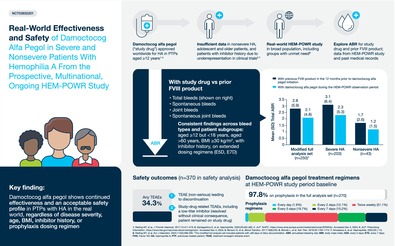

## 1 Introduction

Hemophilia A, an X‐linked, congenital, inherited bleeding disorder, is characterized by deficient coagulation factor VIII (FVIII) [1]. Hemophilia A occurs in more than 400 000 male patients worldwide, with a prevalence of 1 in 5000 males [2, 3]. More recent data suggest that hemophilia is underdiagnosed and estimate that 1 125 000 male patients globally have the disease, with approximately 418 000 being severe [4].

While disease severity based on FVIII activity levels is the most common classification, it has been acknowledged that evaluation of bleeding phenotype and patient preferences, along with disease severity, should guide treatment decisions to individualize care [5]. However, treatment decisions can be challenging for patients with mild and moderate (nonsevere) hemophilia A, due to a lack of bleeding data [6].

Historically, patients with nonsevere disease have been treated on demand while those with severe hemophilia A have typically been treated with prophylaxis [3, 7]. However, there is a growing appreciation that patients with nonsevere disease have poor joint health and also inhibitor development, and hemophilia experts have highlighted the need for more information on appropriate treatment management and further research in nonsevere hemophilia [5, 8, 9]. For patients with moderate hemophilia and a severe phenotype, the World Federation of Hemophilia (WFH) guidelines recommend sufficient prophylaxis to prevent bleeds, with the goal of achieving zero bleeds to avoid any risk of damage [5].

FVIII replacement is given by intravenous infusion and is one of a number of treatment options for hemophilia A [5]. An analysis of pharmacokinetics data on FVIII products for hemophilia A has shown that extended half‐life (EHL) FVIII products have greater area under the curve, indicating greater FVIII levels over time compared to standard half‐life products [10]. Therefore, in addition to providing higher FVIII levels, EHL products may offer flexible dosing regimens, reducing treatment burden and allowing for more individualized therapy based on a patient's lifestyle, activity levels, and age‐related needs [11, 12, 13, 14, 15]. For example, disease management in older patients should consider the risk of comorbidities and surgeries commonly seen in these patients [11, 14]. This differs from younger patients, for whom sufficient bleed protection to sustain a more active lifestyle (e.g., participation in sports) may be a key treatment consideration [16].

Damoctocog alfa pegol (BAY 94‐9027, Jivi®, Bayer) is a B‐domain–deleted EHL recombinant FVIII product that is conjugated in a site‐specific manner with polyethylene glycol (PEGylated) to prolong its half‐life [17, 18]. The PROTECT VIII study (NCT01580293) has demonstrated the efficacy of damoctocog alfa pegol prophylaxis or on‐demand treatment in adult patients with severe hemophilia across different dosing regimens [19]. In the PROTECT VIII extension study, bleeding protection provided by treatment with damoctocog alfa pegol was maintained for more than 5 years, including in patients treated with extended intervals of prophylaxis (every 5 and every 7 days) [20, 21]. Based on data from the pivotal PROTECT VIII studies, damoctocog alfa pegol is approved for use worldwide for the treatment of hemophilia A in previously treated patients (PTPs) aged ≥ 12 years [22, 23, 24, 25, 26].

In clinical trials of FVIII products, including those for damoctocog alfa pegol, patients aged ≥ 60 years, with nonsevere disease, or with a history of FVIII inhibitors are often excluded [19, 27]. Therefore, the findings may not be representative of patients in clinical practice. Additionally, there are limited data specifically for adolescent populations [28]. Further research is particularly important for those with mild hemophilia who are often undertreated and underdiagnosed, and for an aging population whose management of multiple comorbidities is an emerging concern [8, 11, 15]. The same is true for patients with obesity, who may be at increased risk of joint bleeds due to excess weight increasing pressure on the joints and for whom care management is complicated by complex comorbidities [29, 30]. Long‐term, real‐world assessment of FVIII in these subgroups is needed to help inform routine clinical practice.

HEM‐POWR (NCT03932201) is an ongoing, open‐label, real‐world study of damoctocog alfa pegol in PTPs with mild, moderate, and severe hemophilia A. In previous interim analyses, damoctocog alfa pegol has demonstrated bleeding protection and improved joint health, with an acceptable safety profile and tolerability [31]. The objective of the fifth interim analysis of the HEM‐POWR study is to evaluate the effectiveness and safety profile of damoctocog alfa pegol in a larger group of PTPs with severe and nonsevere hemophilia A in a real‐world setting.

## 2 Methods

### 2.1 Study Design

The HEM‐POWR study is a multinational, multicenter, noninterventional, observational, Phase 4 prospective cohort study, evaluating the real‐world effectiveness and safety of damoctocog alfa pegol treatment. A more detailed description of the HEM‐POWR study design was previously published [31, 32]. Study approval was obtained by local independent ethics committees and authorities in participating study centers across Europe, the Americas, and Asia.

The primary objective was annualized bleeding rate (ABR) in PTPs receiving different prophylaxis regimens of damoctocog alfa pegol. The secondary objective was safety and included treatment‐emergent adverse events (TEAEs), serious TEAEs, AEs of special interest, and assessment of hemostasis, which was measured through number of infusions. Baseline demographics and clinical characteristics were collected at study enrollment retrospectively from patient medical records. Patient data collection occurred continuously and was documented through the electronic data capture system. This included the electronic case report form, electronic bleed diary, electronic quality‐of‐life questionnaires, and the AE report form. Reported bleeds were documented in the patient infusion diary, with serious and life‐threatening bleeds documented by the physician.

### 2.2 Study Population

The estimated study completion date is December 31, 2026, and the first patient visit occurred on October 21, 2019. The study included patients aged ≥ 12 years with a previous diagnosis of hemophilia A. PTPs who were damoctocog alfa pegol treatment‐naïve were eligible, as were patients who had initiated or were receiving damoctocog alfa pegol on demand, prophylaxis, or intermittent prophylaxis. Although patients with current evidence or clinical suspicion of FVIII inhibitors were not eligible, patients who had a previous history of FVIII inhibitors and who were on standard prophylaxis therapy for ≥ 1 year prior to enrollment were eligible. Informed consent from patients or their legal representative was required.

Patients who participated in a concurrent program investigating interventions outside of nonclinical practice were excluded. Patients who were diagnosed with any other bleeding/coagulation disorder or received immune tolerance induction treatment at enrollment were also excluded. Patients with contraindications according to local marketing authorization were excluded.

The full analysis set (FAS) was defined as all eligible patients who had a first documented dose of damoctocog alfa pegol, bleeding data, and at least one documented dose in the patient diary. To provide a sufficiently robust characterization of the annualized bleeding data, the modified FAS (mFAS) only included patients from the FAS with ≥ 90 days of observation data documented in the patient diary. The safety analysis set (SAF) was defined as all patients with signed informed consent who received at least one dose of damoctocog alfa pegol during the observation period of the study.

### 2.3 Statistical Methods

Statistical analyses were explorative and descriptive in nature, with no formal hypothesis testing performed. Effectiveness analyses were performed on the mFAS and safety analyses on the SAF. Baseline was defined as the initial visit, and patients were followed up for 180‐day intervals (±90 days). Demographics, baseline characteristics, concomitant disease, inhibitor history, treatment history, and treatment characteristics were described with frequency distributions and/or basic summary statistics for the FAS and SAF. Subgroup data presented in this analysis included patients from the mFAS stratified by disease severity (nonsevere or severe), age (12 to < 18 years or ≥ 60 years), BMI (< 30 kg/m^2^ or ≥ 30 kg/m^2^), bleed type (spontaneous, joint, and spontaneous joint bleeds), and history of FVIII inhibitors (with or without). ABR were reported as mean (standard deviation [SD]) and median (quartile [Q]1, Q3) for 12 months prior to damoctocog alfa pegol initiation and during the observation period. Due to the noninterventional, observational study design, at enrollment patients could be treatment‐naïve or already receiving damoctocog alfa pegol. To allow for a comparison of data before and after initiating damoctocog alfa pegol, the medical history data from prior to enrollment were used for patients already receiving damoctocog alfa pegol (12 months prior to initiation). Further details of the HEM‐POWR study design and analysis are published elsewhere [31, 32].

## 3 Results

In total, 371 patients were enrolled in the HEM‐POWR study at the data cutoff of July 8, 2024. In the SAF and FAS, 370 and 270 patients were included in the analysis, respectively. In the SAF, 1 (0.3%) patient was enrolled and excluded from the analysis as they had not received ≥ 1 dose of damoctocog alfa pegol during the study observation period. In the FAS, 101 (27.2%) patients were excluded from the analysis because they had no documented infusions from the patient diary during the observation period, no documented date of first damoctocog alfa pegol dose in the study, or it was later detected that they were not eligible for inclusion. The mFAS included the 250 patients from the FAS with at least 90 days of diary documentation, starting at Day 1. Baseline is the initial visit; follow‐up windows are defined as half‐year intervals (e.g., follow‐up window 1: days 1–182; follow‐up window 2: days 183–365, etc.).

### 3.1 Baseline Demographics, Characteristics, and Treatment History

As shown in Table S1, in the FAS (*N* = 270), 99.3% of patients were male, 48.2% of patients were white, 79.6% of patients were aged ≥ 18 to < 60 years, and 81.9% of patients had severe hemophilia at initial diagnosis. The median (Q1, Q3) observation period was 764.0 (491.0, 1044.0) days in the SAF and 821.5 (650.0, 1096.0) in the FAS. For patients overall in the FAS, the most common concomitant diseases were chronic pain in 18.5%, hypertension in 16.7%, and human immunodeficiency virus in 10.0%.

All eligible patients were previously treated with a FVIII product, and a large proportion of patients in the FAS were pre‐treated with damoctocog alfa pegol prior to the initial study visit (FAS, 81.1%; 219/270), few of whom had previously participated in a clinical trial of damoctocog alfa pegol (FAS, 8.2%; 18/219). To explore symptoms and treatment characteristics before and after damoctocog alfa pegol initiation, medical history data were used, including data on bleeds and dosing regimen with prior FVIII product (Table 1). The most common dosing regimen with prior FVIII product was every 2 days (Figure S1). Of 317 patients in the SAF and 229 in the FAS for whom data were available on FVIII product prior to the initial study visit, 4.1% and 4.8% received other EHL products, respectively; the remainder received an SHL. One patient received prior non–factor replacement.

**TABLE 1:** Treatment history prior to initial visit and during observation period.

	SAF (*n* = 370)	FAS (*n* = 270)
Medical treatment history with previous FVIII product		
Prophylaxis dosing regimen for previous FVIII product prior to initiation of damoctocog alfa pegol, *n* (%)^a^		
Every day	9 (3.5)	5 (2.7)
Every 2 days	148 (57.4)	103 (55.1)
Twice weekly	83 (32.2)	66 (35.3)
Every 5 days	1 (0.4)	1 (0.5)
Every 6 days	2 (0.8)	1 (0.5)
Every 7 days	12 (4.7)	10 (5.3)
Other	3 (1.2)	1 (0.5)
Annualized total dose per kg for previous FVIII product prior to initiation of damoctocog alfa pegol (IU/kg)^b^		
Median (Q1, Q3)	—	3913.4 (3043.8, 5049.5)
Mean (SD)	—	4335.0 (2227.5)
Medical treatment history prior to enrollment, including patients naïve and pretreated with damoctocog alfa pegol		
Received prophylactic treatment prior to enrollment *n* (%)^c^	345 (93.2)	251 (93.0)
Patients pretreated with damoctocog alfa pegol in the 12 months prior to observation period, *n* (%)	301 (81.4)	219 (81.1)
Most recent modality of damoctocog alfa pegol treatment prior to the observation period, *n* (%)^d^		
Prophylaxis	285 (94.7)	210 (95.9)
On‐demand	13 (4.3)	7 (3.2)
Intermittent prophylaxis	2 (0.7)	2 (0.9)
Treatment with damoctocog alfa pegol during the observation period		
Observation period, days, median (Q1, Q3)	764.0 (491.0, 1044.0)	821.5 (650.0, 1096.0)
Prescribed treatment modality at first dose of damoctocog alfa pegol during the observation period, *n* (%)		
Prophylaxis	356 (96.2)	264 (97.8)
On‐demand	13 (3.5)	5 (1.9)
Intermittent prophylaxis	1 (0.3)	1 (0.4)
Prescribed damoctocog alfa pegol prophylaxis regimen at baseline of the observation period, *n* (%)^e^		
Every day	6 (1.7)	5 (1.9)
Every 2 days	41 (11.5)	32 (12.1)
Twice weekly	187 (52.5)	135 (51.1)
Every 5 days	68 (19.1)	52 (19.7)
Every 7 days	54 (15.2)	40 (15.2)
Patient self‐infusion, yes, *n* (%)	295 (79.7)	219 (81.1)
Annualized total dose per kg for prophylaxis with damoctocog alfa pegol during the observation period (IU/kg)^f^		
Median (Q1, Q3)	—	3345.3 (2452.0, 4290.2)
Mean (SD)	—	3455.3 (2020.4)

Abbreviations: FAS, full analysis set; FVIII, factor VIII; IU, international units; kg, kilogram; Q1, 1st quartile; Q3, 3rd quartile; SAF, safety analysis set; SD, standard deviation.

^a^ Data missing for 112 patients in the SAF and 83 in the FAS.

^b^ Data missing for 96 patients in the FAS.

^c^ Data missing for 3 patients in the SAF and 3 patients in the FAS.

^d^ Data missing for 1 patient in the SAF.

^e^ Patients treated with prophylaxis every day at the baseline of the observation period all had severe hemophilia A.

^f^ Data missing for 5 patients in the FAS.

Baseline characteristics and treatment history data for patient subgroups are summarized in Tables S2 and S3. Most adolescent patients aged 12–< 18 years did not have concomitant disease; hypertension (*n* = 18/28, 64.3%) was the most common for older patients aged ≥ 60 years in the FAS.

### 3.2 Dosing Regimens

The most common dosing regimen during the observation period was twice weekly prophylaxis (Figure S1). Few patients changed their damoctocog alfa pegol treatment regimen during the study; of 239 (88.5%) patients with at least 1 year of follow up, 80.8% (193/239) remained on the same prophylaxis dosing regimen as at baseline. For example, most patients on twice weekly prophylaxis at follow up windows 1 and 2 were on twice weekly prophylaxis at baseline: 129/135 (95.6%) and 119/129 (92.2%), respectively. Similar trends were observed for other dosing regimens; 46/50 (92.0%) and 38/40 (95.0%) patients treated E5D at follow up windows 1 and 2, respectively, were treated E5D at baseline. Likewise, 31/34 (91.2%) and 26/29 (89.7%) patients treated E7D at follow up windows 1 and 2, respectively, were treated E7D at baseline. Similar trends were observed at later follow up windows.

### 3.3 Bleeding in Total Population and Subgroups

**FIGURE 1:**
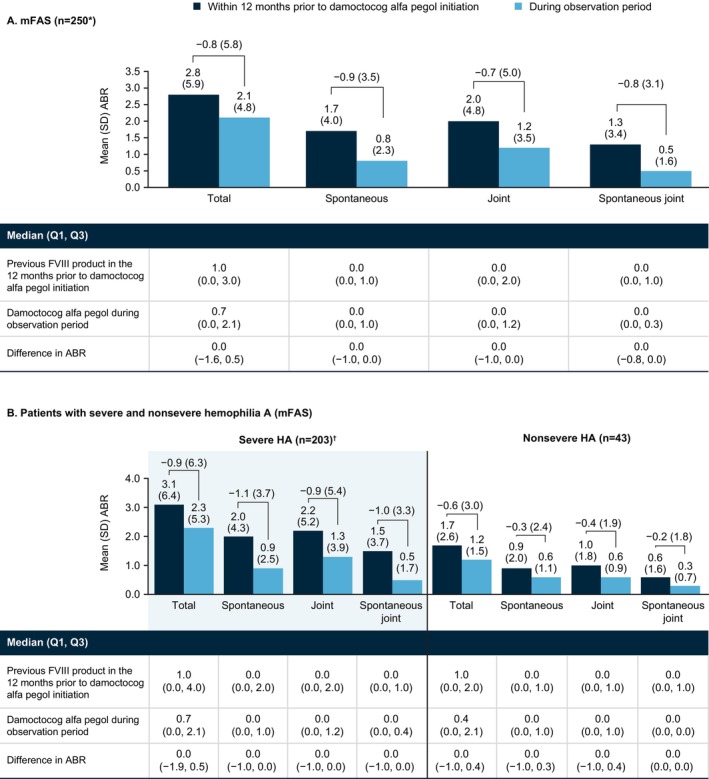
ABR with previous FVIII product in the 12 months prior to damoctocog alfa pegol initiation and with damoctocog alfa pegol during the observation period in the total mFAS (A) and patients stratified by disease severity (B). *Data missing with previous FVIII product prior to damoctocog alfa pegol initiation in 4 patients. ^†^Data missing with previous FVIII product prior to damoctocog alfa pegol initiation in 4 patients. Abbreviations: ABR, annualized bleeding rate; HA, haemophilia A; mFAS, modified full analysis set, including patients with at least 90 days of documentation in the patient diary; Q1, 1st quartile; Q3, 3rd quartile; SD, standard deviation.

In the mFAS (*N* = 250) (Figure 1A), mean (SD)/median ABR for total bleeds was 2.8 (5.9)/1.0 12 months prior to damoctocog alfa pegol initiation and 2.1 (4.8)/0.7 during the observation period, a difference of −0.8 (5.8)/0.0. Similar differences were observed for spontaneous (−0.9 [3.5]/0.0), joint (−0.7 [5.0]/0.0), and spontaneous joint bleeds (−0.8 [3.1]/0.0).

In the disease severity subgroup analysis (Figure 1B), the effectiveness of damoctocog alfa pegol treatment was consistent with that in the total mFAS. In patients with severe (*n* = 203) and nonsevere hemophilia A (*n* = 43), respectively, mean (SD)/median ABR for total bleeds was 3.1 (6.4)/1.0 and 1.7 (2.6)/1.0 12 months prior to damoctocog alfa pegol initiation and 2.3 (5.3)/0.7 and 1.2 (1.5)/0.4 during the observation period, differences of −0.9 (6.3)/0.0 and −0.6 (3.0)/0.0. Similar findings were observed for each bleed type.

**FIGURE 2:**
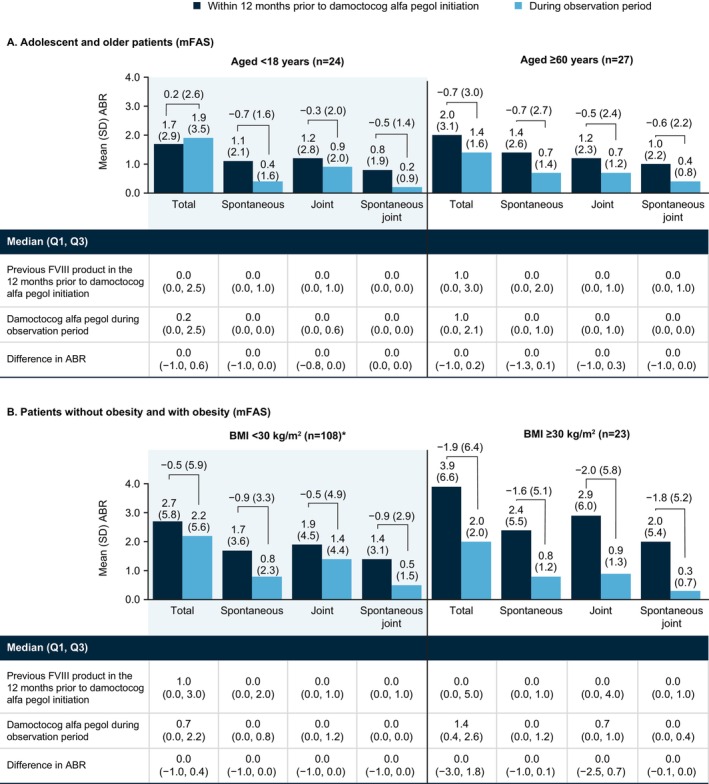
ABR with previous FVIII product in the 12 months prior to damoctocog alfa pegol initiation and with damoctocog alfa pegol during the observation period in mFAS patients stratified by age (A) and BMI (B). *Data missing with previous FVIII product prior to damoctocog alfa pegol initiation in 1 patient. Abbreviations: ABR, annualized bleeding rate; BMI, body mass index; mFAS, modified full analysis set, including patients with at least 90 days of documentation in the patient diary; Q1, 1st quartile; Q3, 3rd quartile; SD, standard deviation.

In adolescents (aged 12 to < 18 years, *n* = 24) (Figure 2A), mean (SD)/median ABR for total bleeds was 1.7 (2.9)/0.0 12 months prior to damoctocog alfa pegol initiation and 1.9 (3.5)/0.2 during the observation period, a difference of 0.2 (2.6)/0.0; across bleed types, differences in ABR were −0.7 (1.6)/0.0 for spontaneous bleeds, −0.3 (2.0)/0.0 for joint bleeds, and −0.5 (1.4)/0.0 for spontaneous joint bleeds. Adolescents did not show a numerical improvement in total bleeds consistent with the total sample. However, this observation appears to be driven by an increase in trauma bleeds during the study since the other bleed types were stable for this subgroup; in adolescents, ABR for trauma bleeds was 0.6 (1.3)/0.0 12 months prior to damoctocog alfa pegol initiation and 1.3 (2.0)/0.0 during the observation period, a difference of 0.8 (1.6)/0.0. In older patients (aged ≥ 60 years, *n* = 27) (Figure 2A), mean (SD)/median ABR for total bleeds was 2.0 (3.1)/1.0 12 months prior to damoctocog alfa pegol initiation and 1.4 (1.6)/1.0 during the observation period, a difference of −0.7 (3.0)/0.0. In this subgroup, results across bleed types were consistent with total bleed data.

In the BMI subgroup analysis (Figure 2B), patients were stratified by obesity (BMI ≥ 30 kg/m^2^, *n* = 23) and non‐obesity (BMI < 30 kg/m^2^, *n* = 108). In patients with obesity and non‐obesity, respectively, mean (SD)/median ABR for total bleeds was 3.9 (6.6)/0.0 and 2.7 (5.8)/1.0 12 months prior to damoctocog alfa pegol initiation and 2.0 (2.0)/1.4 and 2.2 (5.6)/0.7 during the observation period, differences of −1.9 (6.4)/0.0 and −0.5 (5.9)/0.0. Similar findings were observed for each bleed type in each subgroup.

**FIGURE 3:**
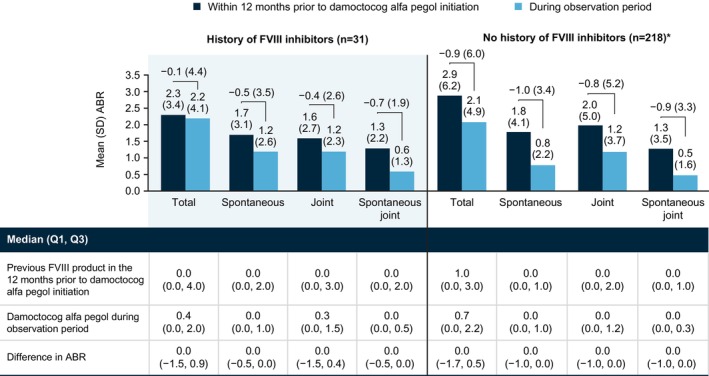
ABR with previous FVIII product in the 12 months prior to damoctocog alfa pegol initiation and with damoctocog alfa pegol during the observation period in mFAS patients stratified by FVIII inhibitor history. *Data missing with previous FVIII product prior to damoctocog alfa pegol initiation in 4 patients. Abbreviations: ABR, annualized bleeding rate; FVIII, factor VIII; mFAS, modified full analysis set, including patients with at least 90 days of documentation in the patient diary; Q1, 1st quartile; Q3, 3rd quartile; SD, standard deviation.

In patients with a history of FVIII inhibitors (*n* = 31) (Figure 3), mean (SD)/median ABR for total bleeds was 2.3 (3.4)/0.0 12 months prior to damoctocog alfa pegol initiation and 2.2 (4.1)/0.4 during the observation period, a difference of −0.1 (4.4)/0.0. Findings were similar across bleed types, with no new inhibitors observed in these patients. In patients with no history of FVIII inhibitors (*n* = 218) (Figure 3), mean (SD)/median ABR for total bleeds was 2.9 (6.2)/1.0 12 months prior to damoctocog alfa pegol initiation and 2.1 (4.9)/0.7 during the observation period, a difference of −0.9 (6.0)/0.0.

**FIGURE 4:**
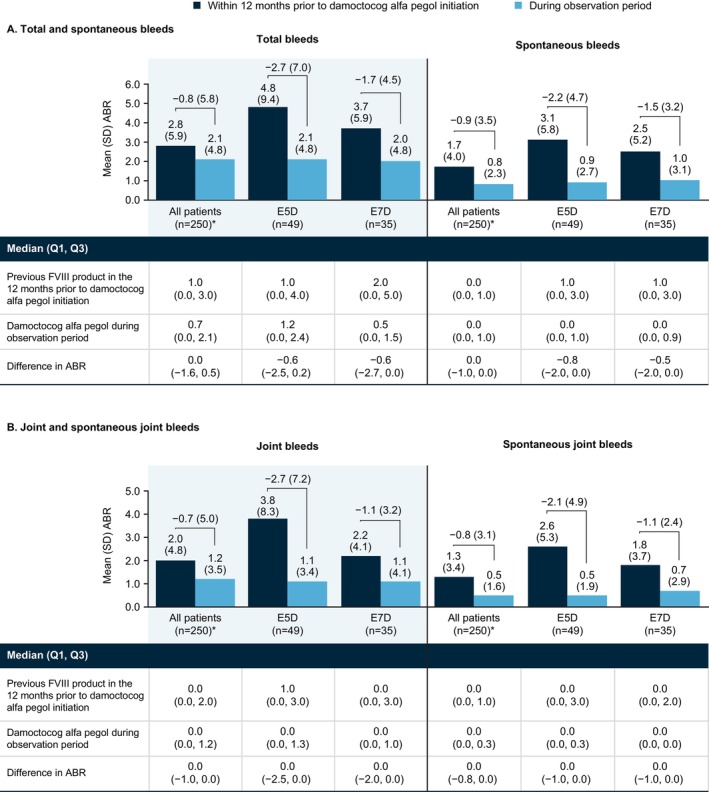
ABR with previous FVIII product in the 12 months prior to damoctocog alfa pegol initiation and with damoctocog alfa pegol during the observation period in mFAS patients treated every 5 days (E5D, *n* = 49) and every 7 days (E7D, *n* = 35), stratified by bleed type. *Data missing with previous FVIII product prior to damoctocog alfa pegol initiation in 4 patients. Abbreviations: ABR, annualized bleeding rate; E5D, every 5 days; E7D, every 7 days; mFAS, modified full analysis set, including patients with at least 90 days of documentation in the patient diary; Q1, 1st quartile; Q3, 3rd quartile; SD, standard deviation.

In patients treated every 5 days (E5D: *n* = 49) and every 7 days (E7D: *n* = 35) (Figure 4), respectively, mean (SD)/median ABR for total bleeds was 4.8 (9.4)/1.0 and 3.7 (5.9)/2.0 12 months prior to damoctocog alfa pegol initiation and 2.1 (4.8)/1.2 and 2.0 (4.8)/0.5 during the observation period, differences of −2.7 (7.0)/−0.6 and −1.7 (4.5)/−0.6. Findings were similar across bleed types for both dosing regimens.

### 3.4 Safety Outcomes

In the SAF, 127/370 (34.3%) patients had TEAEs (Table 2). Three (0.8%) patients reported study‐drug‐related TEAEs: a transient low‐titer inhibitor in a patient with no history of inhibitors (resolved in 4 months without clinical consequence), a haemarthrosis (resolved), and IgG antibodies against FVIII (outcome unknown). One (0.3%) patient reported a TEAE, tendonitis (non‐serious), leading to treatment discontinuation. TEAEs leading to a change in treatment regimen were reported by 42/370 (11.4%) patients; reasons included injury (13/370, 3.5%), musculoskeletal and connective tissue disorders (12/370, 3.2%), and gastrointestinal disorders (7/370, 1.9%). A small number of dose changes were due to increased bleeding frequency (10/196 [5.1%] prescription changes).

**TABLE 2:** Summary of TEAEs.

*n* (%)	SAF (*N* = 370)
Any TEAE	127 (34.3)
Any TEAE leading to a change in treatment regimen	42 (11.4)
Blood and lymphatic system disorders	1 (0.3)
Gastrointestinal disorders	7 (1.9)
Hepatobiliary disorders	1 (0.3)
Infections and infestations	2 (0.5)
Injury and procedural complications	13 (3.5)
Musculoskeletal and connective tissue disorders	12 (3.2)
Renal and urinary disorders	2 (0.5)
Surgical and medical procedures	1 (0.3)
Not coded	9 (2.4)
Any TEAE leading to discontinuation of treatment	1 (0.3)
Any TEAE leading to inhibitor development	1 (0.3)
Any study drug‐related TEAE	3 (0.8)
Any TEAE‐related death	1 (0.3)
Any AESI	4 (1.1)
Inhibitor development/loss of drug effect	1 (0.3)
Low titer FVIII inhibitor	
Neurocognitive disorder	1 (0.3)
Epileptic absence	
Hypersensitivity reaction	1 (0.3)
Erythema on the back	
Renal impairment	1 (0.3)
Urinary incontinence	
Any serious TEAE	42 (11.4)
Any serious TEAE leading to a change in treatment regimen	26 (7.0)
Any serious TEAE leading to discontinuation of treatment	0 (0.0)
Any serious TEAE leading to inhibitor development	1 (0.3)
Any study drug‐related serious TEAE	2 (0.5)
Any serious TEAE‐related death	1 (0.3)

Abbreviations: AESI, adverse events of special interest; FVIII, factor VIII; SAF, safety analysis set; TEAE, treatment‐emergent adverse event.

Four (1.1%) patients reported adverse events of special interest: a transient low‐titer inhibitor in a patient with no history of inhibitors (resolved in 4 months without clinical consequence); petit mal epilepsy (resolved); urinary incontinence (not resolved); and erythema (resolved). Serious TEAEs were observed in 42/370 (11.4%) patients: 34/370 (9.2%) were resolved/resolving, 4/370 (1.1%) were resolved with sequelae, 2 (0.5%) had unknown outcomes, 1 (0.3%) did not resolve, and there was 1 (0.3%) fatality due to spinal cord ischemia, which was determined to be unrelated to the study drug. Of patients with serious TEAEs, 26/370 (7.0%) patients changed treatment regimen as a result.

In the HEM‐POWR study, as may be expected, adolescent patients had few coexisting medical conditions. Joint protection was maintained, and we report an acceptable safety profile in this age group.

## 4 Discussion

The objective of this fifth interim analysis of the HEM‐POWR study was to evaluate the effectiveness and safety profile of damoctocog alfa pegol treatment in PTPs with hemophilia in a real‐world clinical setting in a larger group of patients and over a longer period than previous HEM‐POWR interim analyses. This updated analysis is consistent with previous analyses and demonstrates maintenance of bleeding protection and the acceptable safety profile of damoctocog alfa pegol in patients with hemophilia A [20, 31]. Additionally, with its larger sample enabling subgroup analyses, it provides evidence of maintained effectiveness and acceptable safety across patients stratified by disease severity, age, BMI, patient inhibitor history, and dosing regimen. In patients with a history of FVIII inhibitors, bleeding rates with damoctocog alfa pegol were low, and findings were consistent with the main study data. Importantly, no new inhibitors were seen in these patients; the single inhibitor‐related TEAE observed in the study was transient and reported in a patient with no history of FVIII inhibitors.

Because frequent spontaneous bleeding is more common in severe than in nonsevere disease [8], nonsevere disease can be undertreated, and treatment management may be relatively neglected [15]. In this analysis, the effectiveness of extended dosing regimens in patients with nonsevere hemophilia suggests the viability of regular prophylaxis with an EHL product in patients with mild and moderate disease. There is a lack of data on treatments for patients with nonsevere hemophilia, as clinical trials tend to present research based on severe disease [9]. These real‐world data offer reassurance that safety and effectiveness are maintained for patients taking damoctocog alfa pegol with different treatment schedules depending on their needs. Our findings are novel and can inform patients and clinicians on the treatment management of mild and moderate hemophilia A.

Similar reassurance can be gleaned from the effectiveness data in patients with obesity. Particularly noteworthy is the strong bleeding control for joint and spontaneous joint bleeds, for which high BMI is a risk factor [30]. Moreover, the consistent and compelling reductions in ABR across bleed types suggest the viability of EHL prophylaxis in patients with high BMI despite historic concerns around dosing and self‐administration due to limited venous access [29]. Ultimately, strong bleeding control in patients with obesity, as seen here with damoctocog alfa pegol, may improve global health outcomes through increased activity and better management of complex comorbidities.

Given advances in treatment for hemophilia, there is an aging population of patients [11]. Older patients with hemophilia may have comorbidities (e.g., arthritis, joint damage, cardiovascular disease, liver disease, malignancy, and chronic renal disease) requiring complex treatment pathways to manage care [11, 14, 33]. Individualizing treatment, as with varying dosing regimens, may improve quality of life for these patients [5]. Additionally, older patients with poor venous access may benefit from regimens requiring fewer infusions [34]. Patients who participated in PROTECT VIII exit interviews reported that infusion frequency was one of the most important factors in treatment satisfaction [35]. These patients in PROTECT VIII stated that the longer duration of factor coverage and less frequent infusions with damoctocog alfa pegol were associated with better vein health and reduced impact on activities and work [35]. Therefore, it is important for healthcare professionals to understand the patient's lifestyle to individualize their treatment, and for patients to have shared decision‐making in their care [36].

The same is true for adolescent patients, who may have other healthcare priorities driving treatment decisions, such as increased activity levels compared with older patients [16]. Additionally, adolescents are navigating the transition to independent treatment management, as they become adults. Our results indicate that damoctocog alfa pegol can offer flexible dosing regimens, with most patients being treated twice weekly with damoctocog alfa pegol during the observation period versus every 2 days with their previous FVIII product. Flexible dosing regimens may enable an active lifestyle for adolescents and make it simpler for them to manage their own care as they transition into adulthood [16].

In the current study, total ABR for adolescents was higher with damoctocog alfa pegol during the observation period than with the previous FVIII product prior to damoctocog alfa pegol initiation. However, this increase was driven by a rise in trauma bleeds, which may indicate better global bleed control leading to greater physical activity; Shrestha et al. found that good bleed control can have a positive impact on physical activity levels in young people and adults with hemophilia A. Therefore, it is possible that adolescents in the current study may have engaged in more physical activity as their spontaneous and joint bleeds decreased, potentially leading to a rise in trauma bleeds. These findings may be generalizable to patients of all ages and with different disease characteristics who wish to participate in activities or sports.

Limitations and strengths of the HEM‐POWR study have been previously discussed [31]. Key limitations include the study's noninterventional design and observational nature, which may give rise to bias through patient selection and bleeding event recall, and limitations on the availability of historical medical record data. Because recruitment is not balanced across countries, country‐specific prescribing practices, such as differences in local guidelines, may likewise result in bias. A further limitation is smaller subgroup sample sizes, inherent to patient stratification (e.g., for adolescents [*n* = 24] or patients with obesity [*n* = 23]); therefore, findings from subgroup analyses should be interpreted with caution.

To our knowledge, to date, the HEM‐POWR study is the largest prospective real‐world study of EHL FVIII products including a patient population from multiple centers and countries, with a long observation period. Patients enrolled in this study reflect the real‐world clinical setting and include those who may be excluded from clinical trials, specifically older patients and those with a history of FVIII inhibitors. While clinical trial data are essential to provide robust findings on the efficacy and safety of a drug, real‐world data are also crucial to provide valuable insights into effectiveness in a patient population with a broader medical history.

## 5 Conclusions

This fifth interim analysis further supports the effectiveness and acceptable safety profile of damoctocog alfa pegol for the treatment of PTPs with severe or nonsevere hemophilia A. In this real‐world study, damoctocog alfa pegol maintained bleed protection and was well tolerated with no new safety concerns related to the study drug after follow‐up. The broad, real‐world patient population included in the HEM‐POWR study will help inform patients, clinicians, and stakeholders on the routine clinical use of damoctocog alfa pegol in hemophilia A.

## Author Contributions

Mark T. Reding, María Teresa Alvarez Román, Giancarlo Castaman, Maissaa Janbain, Tadashi Matsushita, Karina Meijer, and Johannes Oldenburg were principal investigators who treated patients and contributed to data acquisition and interpretation. Kathrin Schmidt was the clinical project manager, responsible for the conduct of the study and contribution to data analysis and interpretation. All authors contributed to the development of the manuscript and approved the final draft.

## Conflicts of Interest

M.T.R.: receipt of institutional research support from Bayer, BioMarin and Be Bio, member of advisory boards and/or speaker bureaus for Bayer, CSL Behring, Genentech, HEMA Biologics, Novo Nordisk, Sanofi, Spark and Takeda; M.T.A.R.: speaker in advisory boards and sponsored symposia with Novo Nordisk, Bayer, Takeda, Roche, Pfizer, Octapharma, Amgen, Novartis, CSL Behring and Sobi; G.C.: speaker at satellite symposia during scientific meetings for Bayer, Grifols, LFB, Roche, Sobi, Novo Nordisk, Werfen and Kedrion, member of steering committee of UniQure, participant of advisory boards for Alexion, Bayer, BioMarin, Takeda, CSL Behring, LFB, Novo Nordisk, Pfizer, Roche, Sanofi, Sobi and UniQure, consultant for Roche; M.J.: member of speaker bureau for Takeda, BioMarin, CSL Behring and Sanofi, consultancy for Takeda, CSL Behring, Sanofi, BioMarin, Genentech and Octapharma, member of Bayer steering committee; T.M.: member of advisory boards for Takeda, Bayer, Novo Nordisk, Chugai and Pfizer, receipt of educational and investigational support from Chugai and Novo Nordisk, received honoraria from Takeda, Bayer, Sanofi, Chugai, CSL Behring, JB Pharma, KMB Pharma, Novo Nordisk, Octapharma and Sysmex; K.M.: receipt of speaker fees from Alexion, participation in trial steering committees for Bayer and Astra Zeneca, consulting fees from Therini, participation in data monitoring and endpoint adjudication committee for Octapharma; K.S.: employee of Bayer; J.O.: reimbursed for attending symposia/congresses and/or received honoraria and/or funds for research from Bayer, Biogen Idec, Biomarin, Biotest, Chugai, CSL Behring, Freeline, Grifols, LFB, Novo Nordisk, Octapharma, Pfizer, Roche, Sanofi, Spark Therapeutics, Swedish Orphan Biovitrum and Takeda.

## Supporting information


**Data S1:** Supporting Information.

**Video S1:** Video summary of the real‐world HEM POWR study interim 5 results.

## Data Availability

Availability of the data underlying this publication will be determined according to Bayer's commitment to the EFPIA/PhRMA “Principles for responsible clinical trial data sharing.” This pertains to scope, timepoint, and process of data access. As such, Bayer commits to sharing upon request from qualified scientific and medical researchers, patient‐level clinical trial data, study‐level clinical trial data, and protocols from clinical trials in patients for medicines and indications approved in the United States (US) and European Union (EU) as necessary for conducting legitimate research. This applies to data on new medicines and indications that have been approved by the EU and US regulatory agencies on or after January 01, 2014. Interested researchers can use www.vivli.org to request access to anonymized patient‐level data and supporting documents from clinical studies to conduct further research that can help advance medical science or improve patient care. Information on the Bayer criteria for listing studies and other relevant information is provided in the member section of the portal. Data access will be granted to anonymized patient‐level data, protocols, and clinical study reports after approval by an independent scientific review panel. Bayer is not involved in the decisions made by the independent review panel. Bayer will take all necessary measures to ensure that patient privacy is safeguarded.
