# Supplementary material for: Real‐World Effectiveness and Safety of Damoctocog Alfa Pegol in Severe and Nonsevere Patients With Hemophilia A From the Prospective, Multinational, Ongoing HEM‐POWR Study
Source: Eur J Haematol. 2025 Oct 22;116(2):148–59. doi: 10.1111/ejh.70026 (PMC12781148; doi:10.1111/ejh.70026)
Supplement: Supplementary file 1 — Data S1: Supporting Information. [file EJH-116-148-s002.docx]

Real-World Effectiveness and Safety of Damoctocog Alfa Pegol in Severe and Nonsevere Patients With Hemophilia A From the Prospective, Multinational, Ongoing HEM-POWR Study

**Authors**: Mark T Reding^1^, María Teresa Alvarez Román^2^, Giancarlo Castaman^3^, Maissaa Janbain^4^, Tadashi Matsushita^5^, Karina Meijer^6^, Kathrin Schmidt^7^, Johannes Oldenburg^8^

**Affiliations**:

^1^Center for Bleeding and Clotting Disorders, University of Minnesota Medical Center, Minneapolis, Minnesota, USA; redin002@umn.edu

^2^Thrombosis and Haemostasis Unit, Hospital Universitario La Paz, Madrid, Spain; talvarezroman@gmail.com

^3^Department of Oncology, Center for Bleeding Disorders and Coagulation, Careggi University Hospital, Florence, Italy; castaman@aou-careggi.toscana.it

^4^Deming Department of Internal Medicine, Section of Hematology and Medical Oncology, Tulane School of Medicine, New Orleans, Louisiana, USA; mjanbain@tulane.edu

^5^Department of Transfusion Medicine, Nagoya University Hospital, Nagoya, Japan; tmatsu@med.nagoya-u.ac.jp

^6^Department of Hematology, University Medical Center Groningen, Groningen, Netherlands; k.meijer@umcg.nl

^7^OS Operations, Bayer, Berlin, Germany; kathrin.schmidt@bayer.com

^8^Institute of Experimental Hematology and Transfusion Medicine, University Hospital Bonn, Medical Faculty, University of Bonn, Bonn, Germany; johannes.oldenburg@ukbonn.de

Corresponding author: *Dr Mark T Reding*

**Table S1**. Baseline demographics and characteristics

|  | **SAF (n=370)** | **FAS (n=270)** |
| --- | --- | --- |
| Male, n (%) | 368 (99.5) | 268 (99.3) |
| Race, n (%)* |  |  |
| Asian | 87 (23.5) | 82 (30.4) |
| White | 197 (53.2) | 130 (48.2) |
| Black or African American | 9 (2.4) | 7 (2.6) |
| American Indian or Alaska Native | 0 | 0 |
| Native Hawaiian or other Pacific Islander | 1 (0.3) | 1 (0.4) |
| Not reported | 27 (7.3) | 21 (7.8) |
| Age at enrollment, years, n (%) |  |  |
| ≥12 to <18 | 40 (10.8) | 27 (10.0) |
| ≥18 to <60 | 296 (80.0) | 215 (79.6) |
| ≥60 | 34 (9.2) | 28 (10.4) |
| Weight at baseline, kg, median (min, max)^†^ | 80.0 (44.0, 185.0) | 79.0 (44.0, 185.0) |
| BMI at baseline, kg/m^2^, median (min, max)^‡^ | 25.0 (16.8, 53.2) | 25.0 (16.8, 53.2) |
| Country, n (%) |  |  |
| Japan | 61 (16.5) | 59 (21.9) |
| Germany | 73 (19.7) | 54 (20.0) |
| USA | 31 (8.4) | 27 (10.0) |
| Greece | 18 (4.9) | 4 (1.5) |
| Norway | 18 (4.9) | 0 |
| Denmark | 22 (6.0) | 21 (7.8) |
| Canada | 25 (6.8) | 24 (8.9) |
| Italy | 58 (15.7) | 28 (10.4) |
| Taiwan  Other^§^ | 20 (5.4)  44 (11.9) | 18 (6.7)  35 (13.0) |
| Most common concomitant disease, n (%) |  |  |
| Chronic pain  Hypertension | 70 (18.9)  56 (15.1) | 50 (18.5)  45 (16.7) |
| HIV test positive | 31 (8.4) | 27 (10.0) |
| Liver disease  HCV test positive | 17 (4.6)  23 (6.2) | 14 (5.2)  13 (4.8) |
| Family history of hemophilia, yes, n (%)^¶^ | 196 (53.0) | 143 (53.0) |
| Severity of hemophilia at initial diagnosis, n (%)** |  |  |
| Mild | 12 (3.2) | 6 (2.2) |
| Moderate | 52 (14.1) | 39 (14.4) |
| Severe | 302 (81.6) | 221 (81.9) |
| Patient history of inhibitors, yes, n (%)^††^ | 48 (13.0) | 35 (13.0) |
| Immune tolerance induction history, yes, n (%) | 24 (6.5) | 16 (5.9) |

*Data missing for 49 patients in the SAF and 29 in the FAS; ^†^data missing for 138 patients in the SAF and 103 in the FAS; ^‡^data missing for 171 in patients in the SAF and 128 in the FAS; ^§^Belgium, Brazil, Colombia, Netherlands, Slovenia, Spain, and Sweden; ^¶^data missing from 16 in patients in the SAF and 12 in the FAS; **data missing for 4 patients in the SAF and FAS; ^††^data missing for 1 patient in the SAF and FAS.

Abbreviations: BMI, body mass index; FAS; full analysis set; HCV, hepatitis C virus; HIV, human immunodeficiency virus; kg, kilogram; m, meter; max, maximum; min, minimum; Q1, 1^st^ quartile; Q3, 3^rd^ quartile; SAF, safety analysis set.

**Table S2**. Baseline demographics and treatment characteristics for patients in the FAS stratified by age and BMI

|  | **Aged 12 to <18 years** (n=27) | **Aged ≥60 years**  (n=28) | **BMI <30 kg/m^2^**  (n=118) | **BMI ≥30 kg/m^2^**  (n=24) |
| --- | --- | --- | --- | --- |
| Male, n (%) | 27 (100.0) | 27 (96.4) | NR | NR |
| Severity of hemophilia at initial diagnosis, n (%)  Mild or moderate  Severe  Missing | 4 (14.8)  23 (85.2)  0 | 12 (42.9)  14 (50.0)  2 (7.1) | 22 (18.6)  95 (80.5)  1 (0.8) | 8 (33.3)  16 (66.7)  0 |
| Patient history of inhibitors, yes, n (%) | 4 (14.8) | 1 (3.6) | 14 (11.9) | 2 (8.3) |
| Most recent previous FVIII treatment modality prior to damoctocog alfa pegol initiation, n (%)  On demand  Prophylaxis  Missing | 3 (11.1)  23 (85.2)  1 (3.7) | 9 (32.1)  16 (57.1)  3 (10.7) | 13 (11.0)  93 (78.8)  12 (10.2) | 5 (20.8)  15 (62.5)  4 (16.7) |
| Prescribed prophylaxis regimen for previous FVIII product prior to damoctocog alfa pegol initiation, n (%)  Every day  Every 2 days  Twice weekly  Every 5 days  Every 6 days  Every 7 days  Other | 0  17 (81.0)  3 (14.3)  1 (4.8)  0  0  0 | 1 (6.7)  4 (26.7)  9 (60.0)  0  0  1 (6.7)  0 | 2 (2.3)  53 (60.2)  29 (33.0)  1 (1.1)  0  3 (3.4)  0 | 1 (6.7)  11 (73.3)  3 (20.0)  0  0  0  0 |
| Prescribed prophylaxis regimen with damoctocog alfa pegol at baseline of the observation period, n (%)  Every day  Every 2 days  Twice weekly  Every 5 days  Every 7 days  Other | 0  3 (11.5)  16 (61.5)  6 (23.1)  1 (3.9)  0 | 1 (4.2)  0  12 (50.0)  3 (12.5)  8 (33.3)  0 | 2 (1.7)  14 (12.0)  61 (52.1)  26 (22.2)  14 (12.0)  0 | 1 (4.4)  2 (8.7)  10 (43.5)  8 (34.8)  2 (8.7)  0 |

Abbreviations: BMI, body mass index; FAS; full analysis set; NR, not reported.

**Table S3**. Baseline demographics and treatment characteristics for patients in the FAS stratified by inhibitor history, disease severity, and dosing regimen

|  | **Inhibitor history** | | **Disease severity** | | **Dosing regimen** | |
| --- | --- | --- | --- | --- | --- | --- |
|  | **Yes**  (n=35) | **No**  (n=234) | **Mild, moderate**  (n=45) | **Severe**  (n=221) | **Every 5 days**  (n=52) | **Every 7 days**  (n=40) |
| Male, n (%) | 35 (100.0) | 233 (99.6) | 45 (100.0) | 221 (100.0) | 52 (100.0) | 40 (100.0) |
| Race, n (%)  White  Black or African American  Asian  American Indian or Alaska Native  Native Hawaiian or other Pacific Islander  Not reported  Missing | 17 (48.6)  0  11 (31.4)  0  0  2 (5.7)  5 (14.3) | 113 (48.3)  7 (3.0)  70 (29.9)  0  1 (0.4)  19 (8.1)  24 (10.3) | 21 (46.7)  0  8 (17.8)  0  1 (2.2)  7 (15.6)  8 (17.8) | 107 (48.4)  7 (3.2)  73 (33.0)  0  0  13 (5.9)  21 (9.5) | 29 (55.8)  2 (3.9)  15 (28.9)  0  0  1 (1.9)  5 (9.6) | 13 (32.5)  0  20 (50.0)  0  1 (2.5)  3 (7.5)  3 (7.5) |
| Country, n (%)  Belgium  Brazil  Canada  Colombia  Denmark  Germany  Greece  Italy  Japan  Netherlands  Norway  Slovenia  Spain  Sweden  Taiwan  USA | 0  0  1 (2.9)  2 (5.7)  3 (8.6)  13 (37.1)  1 (2.9)  0  8 (22.9)  0  0  0  1 (2.9)  2 (5.7)  3 (8.6)  1 (2.9) | 1 (0.4)  6 (2.6)  23 (9.8)  4 (1.7)  18 (7.7)  41 (17.5)  3 (1.3)  28 (12.0)  51 (21.8)  2 (0.9)  0  3 (1.3)  8 (3.4)  6 (2.6)  15 (6.4)  25 (10.7) | 0  0  3 (6.7)  2 (4.4)  6 (13.3)  10 (22.2)  2 (4.4)  5 (11.1)  6 (13.3)  0  0  0  1 (2.2)  2 (4.4)  1 (2.2)  7 (15.6) | 1 (0.5)  6 (2.7)  21 (9.5)  4 (1.8)  15 (6.8)  44 (19.9)  2 (0.9)  22 (10.0)  53 (24.0)  2 (0.9)  0  3 (1.4)  8 (3.6)  6 (2.7)  17 (7.7)  17 (7.7) | 0  2 (3.9)  1 (1.9)  0  4 (7.7)  11 (21.2)  0  8 (15.4)  4 (7.7)  0  0  1 (1.9)  2 (3.9)  1 (1.9)  9 (17.3)  9 (17.3) | 0  0  1 (2.5)  0  2 (5.0)  6 (15.0)  1 (2.5)  4 (10.0)  16 (40.0)  0  0  0  3 (7.5)  1 (2.5)  4 (10.0)  2 (5.0) |
| *Table continues on next page* |  |  |  |  |  |  |
|  |  |  |  |  |  |  |
|  | **Inhibitor history** | | **Disease severity** | | **Dosing regimen** | |
|  | **Yes**  (n=35) | **No**  (n=234) | **Mild, moderate**  (n=45) | **Severe**  (n=221) | **Every 5 days**  (n=52) | **Every 7 days**  (n=40) |
| Age at enrollment, years, n (%)  ≥12 to <18  ≥18 to <60  ≥60 | 4 (11.4)  30 (85.7)  1 (2.9) | 23 (9.8)  184 (78.6)  27 (11.5) | 4 (8.9)  29 (64.4)  12 (26.7) | 23 (10.4)  184 (83.3)  14 (6.3) | 6 (11.5)  43 (82.7)  3 (5.8) | 1 (2.5)  31 (77.5)  8 (20.0) |
| BMI at baseline, kg/m^2^, median (min, max)* | 23.3 (18.7, 36.4) | 25.0 (16.8, 53.2) | 25.8 (16.8, 39.0) | 24.9 (16.8, 53.2) | 25.7 (16.8, 49.9) | 23.5 (18.7, 33.6) |
| Family history of hemophilia, yes, n (%)^†^ | 18 (51.4) | 124 (53.0) | 24 (53.3) | 116 (52.5) | 33 (63.5) | 15 (37.5) |
| Severity of hemophilia at initial diagnosis, n (%)  Mild  Moderate  Severe  Missing | 0  1 (2.9)  34 (97.1)  0 | 6 (2.6)  38 (16.2)  187 (79.9)  3 (1.3) | 6 (13.3)  39 (86.7)  0  0 | 0  0  221 (100.0)  0 | 1 (1.9)  5 (9.6)  45 (86.5)  1 (1.9) | 4 (10.0)  12 (30.0)  24 (60.0)  0 |
| Patient history of inhibitors, yes, n (%) | 35 (100.0) | 0 | 1 (2.2) | 34 (15.4) | 3 (5.8) | 5 (12.5) |
| Most recent previous FVIII treatment modality prior to damoctocog alfa pegol initiation, n (%)  On demand  Prophylaxis  Missing | 3 (8.6)  27 (77.1)  5 (14.3) | 31 (13.3)  165 (70.5)  38 (16.2) | 19 (42.2)  22 (48.9)  4 (8.9) | 15 (6.8)  169 (76.5)  37 (16.7) | 8 (15.4)  38 (73.1)  6 (11.5) | 15 (37.5)  20 (50.0)  5 (12.5) |
| Prescribed prophylaxis regimen for previous FVIII product prior to damoctocog alfa pegol initiation, n (%)  Every day  Every 2 days  Twice weekly  Every 5 days  Every 6 days  Every 7 days  Other | 3 (11.1)  20 (74.1)  4 (14.8)  0  0  0  0 | 2 (1.3)  83 (51.9)  62 (38.8)  1 (0.6)  1 (0.6)  10 (6.3)  1 (0.6) | 1 (4.8)  8 (38.1)  10 (47.6)  0  0  2 (9.5)  0 | 4 (2.4)  95 (57.6)  55 (33.3)  1 (0.6)  1 (0.6)  8 (4.8)  1 (0.6) | 1 (2.7)  20 (54.1)  13 (35.1)  1 (2.7)  1 (2.7)  1 (2.7)  0 | 1 (5.0)  2 (10.0)  9 (45.0)  0  0  7 (35.0)  1 (5.0) |
| *Table continues on next page* |  |  |  |  |  |  |
|  |  |  |  |  |  |  |
|  | **Inhibitor history** | | **Disease severity** | | **Dosing regimen** | |
|  | **Yes**  (n=35) | **No**  (n=234) | **Mild, moderate**  (n=45) | **Severe**  (n=221) | **Every 5 days**  (n=52) | **Every 7 days**  (n=40) |
| Prescribed prophylaxis regimen with damoctocog alfa pegol at baseline of the observation period, n (%)  Every day  Every 2 days  Twice weekly  Every 5 days  Every 7 days | 3 (8.8)  10 (29.4)  13 (38.2)  3 (8.8)  5 (14.7) | 2 (0.9)  22 (9.6)  121 (52.8)  49 (21.4)  35 (15.3) | 0  2 (4.9)  17 (41.5)  6 (14.6)  16 (39.0) | 5 (2.3)  30 (13.6)  116 (52.7)  45 (20.5)  24 (10.9) | 0  0  0  52 (100.0)  0 | 0  0  0  0  40 (100.0) |

*Data missing for 19 patients with a history of inhibitors, 108 patients without a history of inhibitors, 15 patients with nonsevere disease, 110 patients with severe disease, 18 patients treated E5D, and 24 patients treated E7D. ^†^Data missing for 12 patients without a history of inhibitors, 2 patients with nonsevere disease, 10 patients with severe disease, 2 patients treated E5D, and 3 patients treated E7D.

Abbreviations: BMI, body mass index; FAS; full analysis set; FVIII, factor VIII.

**Figure S1**. Prophylaxis dosing regimen with previous FVIII product in the 12 months prior to damoctocog alfa pegol initiation and with damoctocog alfa pegol during the observation period for patients in the FAS (n=270)


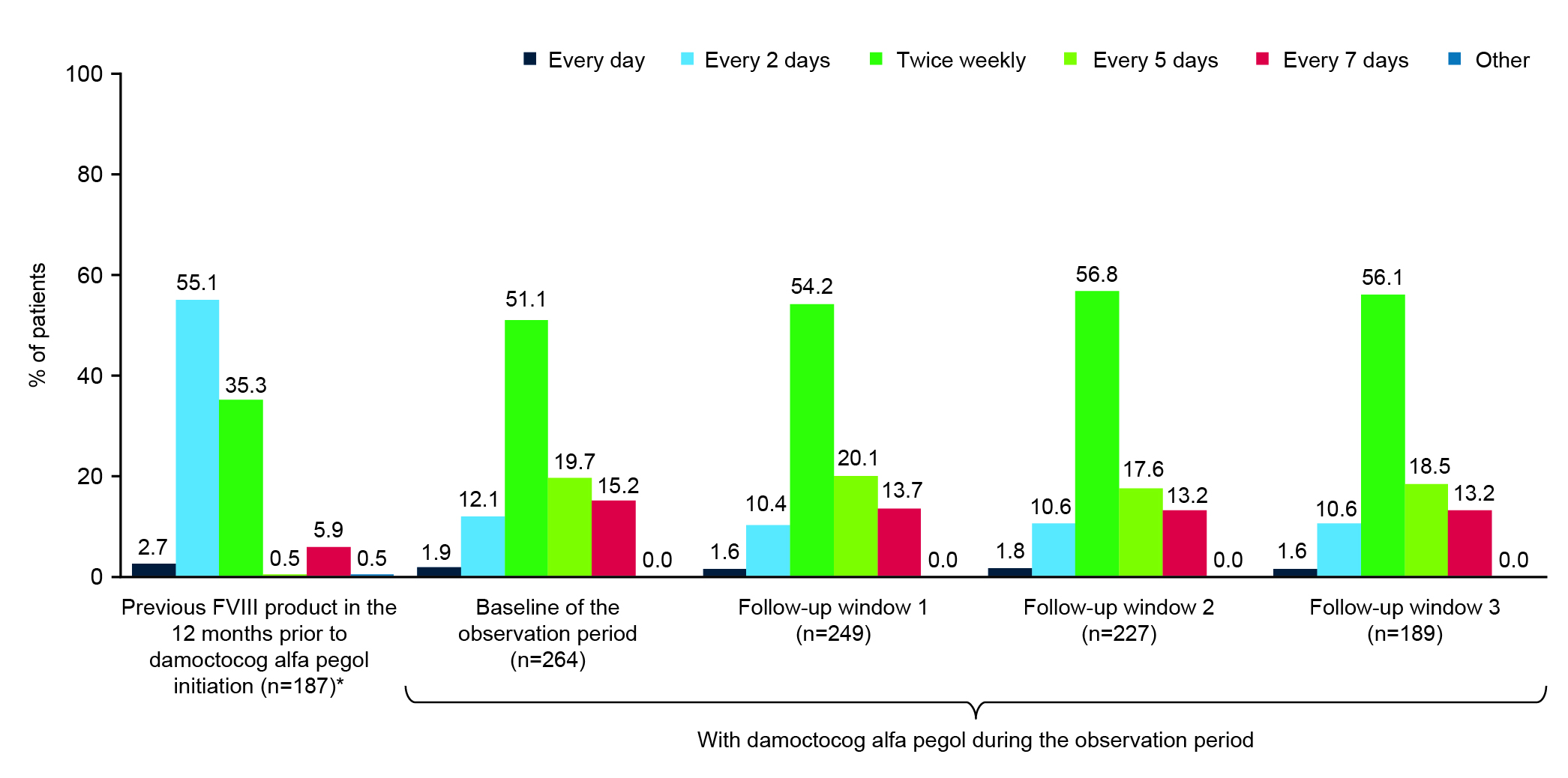


*Regimen E7D includes 1 (0.5%) patient on E6D. Follow-up windows are defined as half-year intervals. Follow-up window 1: days 1 - 182, Follow-up window 2: days 183 - 365, etc.

Abbreviations: FAS, full analysis set; FVIII, factor VIII.
